# Development of a combined GMMA and glycoconjugate iNTS-TCV trivalent vaccine against invasive salmonellosis

**DOI:** 10.21203/rs.3.rs-10957216/v1

**Published:** 2026-10-04

**Authors:** Francesco Citiulo, Francesca Necchi, Emilia Cappelletti, Xhenti Ferhati, Maria Grazia Aruta, Ilaria Liverani, Chiara Muzzi, Marta Benincasa, Carlo Giannelli, Francesca Mancini, Omar Rossi, Francesca Micoli, Claudia G. Vitali, Laura B. Martin, Francesco Berlanda Scorza, Anna Maria Colucci, Rocío Canals

**Affiliations:** GSK Vaccines Institute for Global Health S.r.l. (GVGH); GSK Vaccines Institute for Global Health S.r.l. (GVGH); GSK Vaccines Institute for Global Health S.r.l. (GVGH); GSK Vaccines Institute for Global Health S.r.l. (GVGH); GSK Vaccines Institute for Global Health S.r.l. (GVGH); GSK Vaccines Institute for Global Health S.r.l. (GVGH); GSK Vaccines Institute for Global Health S.r.l. (GVGH); GSK Vaccines Institute for Global Health S.r.l. (GVGH); GSK Vaccines Institute for Global Health S.r.l. (GVGH); GSK Vaccines Institute for Global Health S.r.l. (GVGH); GSK Vaccines Institute for Global Health S.r.l. (GVGH); GSK Vaccines; GSK Vaccines; GSK Vaccines Institute for Global Health S.r.l. (GVGH); GSK Vaccines; GSK Vaccines Institute for Global Health S.r.l. (GVGH); GSK Vaccines Institute for Global Health S.r.l. (GVGH)

**Keywords:** Salmonella, invasive nontyphoidal Salmonella (iNTS), GMMA, typhoid conjugate vaccine (TCV)

## Abstract

Non-typhoidal *Salmonella* (NTS) is a leading cause of bloodstream infections associated with substantial mortality mostly in sub-Saharan Africa (sSA) in children under 5 years. *Salmonella enterica* serovars Typhimurium and Enteritidis account for more than 90% of invasive NTS (iNTS) infections. Typhoid fever is caused by *S. enterica* serovar Typhi and is relatively common in South and Southeast Asia and sSA, especially among children. While there are licensed vaccines to prevent *S*. Typhi infections, the only current intervention for iNTS disease is treatment with antibiotics.

This work presents the preclinical development of a trivalent vaccine, iNTS-TCV, to prevent both iNTS disease and typhoid fever. This vaccine combines the Generalized Modules for Membrane Antigens (GMMA) technology for the iNTS components with the WHO-pre-qualified Vi-CRM_197_ Typhoid Conjugate Vaccine (TCV). Immunization of mice with the iNTS-TCV candidate vaccine elicited higher anti-Vi IgG responses, characterized by a distinct IgG subclass profile, and greater serum bactericidal activity compared to responses induced by Vi-CRM_197_ alone. *In vitro* immunoprofiling of human peripheral blood mononuclear cells revealed that the GMMA components drive a robust pro-inflammatory cytokine response, suggesting a potential mechanistic basis for the observed adjuvant effect on Vi-CRM_197_ immunogenicity. The iNTS-TCV vaccine was well-tolerated in preclinical toxicology studies.

These findings demonstrate the feasibility and potential benefits of combining GMMA and glycoconjugate vaccine technologies, offering a promising strategy for the prevention of both iNTS disease and typhoid fever. This trivalent vaccine approach may provide a sustainable and effective solution for addressing the significant burden of invasive salmonellosis in sSA.

## Introduction

Invasive non-typhoidal *Salmonella* (iNTS) disease and typhoid fever pose a substantial and persistent threat to global public health, particularly within low- and middle-income countries (LMICs)^1,2^. iNTS disease, predominantly caused by *Salmonella enterica* serovars Typhimurium (*S*. Typhimurium) and Enteritidis (*S*. Enteritidis), was responsible for an estimated 509,976 incident cases and 62,018 deaths worldwide in 2021, with infants and young children, especially those under five years of age, in sub-Saharan Africa (sSA) experiencing the highest burden^3^. The pooled case-fatality ratio for iNTS disease is alarmingly high, estimated 17% in Africa^4^. This high mortality is often exacerbated by rapid disease progression, non-specific clinical symptoms leading to misdiagnosis, and limited access to timely, confirmatory blood culture diagnostics in resource-constrained settings^5^. This disease is associated with host risk factors prevalent in sSA, including malaria, acute malnutrition and HIV infection^5^. Moreover, *Salmonella* strains causing iNTS are associated with increasing prevalence of multidrug resistance (MDR), including resistance to extended-spectrum cephalosporins and fluoroquinolones^6^, severely limiting effective treatment options and making vaccine development a global health need^7,8^. The causative agent of typhoid fever is *S. enterica* serovar Typhi (*S*. Typhi), which is primarily transmitted through contaminated food and water. Typhoid fever remains a significant global public health problem, with an estimated 7.2 million cases and 93,333 deaths in 2021 worldwide^9^. Children are disproportionately affected, with the highest incidence rates observed in the 5–9 year age group^10^. The persistent high burden of both iNTS disease and typhoid fever, coupled with the growing threat of antimicrobial resistance, underscores the critical need for comprehensive vaccine solutions.

Despite the high global disease burden, particularly in sSA, there is currently no licensed vaccine available against iNTS disease, while typhoid conjugate vaccines (TCVs) have been successfully developed and are recommended by the World Health Organization (WHO)^11^. To address invasive salmonellosis, the development of combination vaccines that can target multiple *Salmonella* serovars simultaneously has emerged as a promising strategy. These multivalent vaccines aim to enhance programmatic efficiency and potentially reduce costs associated with manufacturing and distribution compared to standalone options^12,13^.

One vaccine candidate developed by GSK Vaccines Institute for Global Health (GVGH) and currently in clinical development is a trivalent vaccine designed to prevent both iNTS disease and typhoid fever. This vaccine, referred to as iNTS-TCV, combines antigens from the two most prevalent iNTS-causing *S. enterica* serovars, Typhimurium and Enteritidis, with a typhoid conjugate vaccine component to provide additional protection against *S*. Typhi^8^. The iNTS component of this vaccine leverages Generalized Modules for Membrane Antigens (GMMA) technology^14^. GMMA are outer membrane vesicles derived from genetically modified bacteria, allowing for the presentation of polysaccharide and multiple membrane protein antigens in their natural membrane environment, thereby enhancing immunogenicity while reducing the risk of systemic reactogenicity^15^. The trivalent formulation of iNTS-TCV specifically combines the bivalent iNTS-GMMA component^16^ with a WHO-prequalified TCV, which is a Vi-CRM_197_ glycoconjugate vaccine^17^. The O-antigen (OAg) portion of the lipopolysaccharide (LPS) is a key target antigen for immunity against *Salmonella*, and GMMA are effective delivery systems for these OAg components^18^. *S*. Typhimurium and *S*. Enteritidis belong to serogroups O:4 and O:9, respectively, which together account for 90% of iNTS infections^19^; therefore, a vaccine targeting these serogroups could have the potential to prevent the vast majority of iNTS disease^20^. Preclinical studies evaluating a bivalent iNTS-GMMA formulation in both mice and rabbits have demonstrated its ability to elicit antibodies capable of complement-mediated killing against a broad panel of clinically relevant *Salmonella* strains^21^. Based on preclinical results in mice and rabbits, we have demonstrated that Alhydrogel does not increase the IgG and functional (SBA) response induced by GMMA^18,22^, but GMMA have been tested in clinical trials adsorbed on aluminum hydroxide to potentially enhance their tolerability^23^.

The Vi antigen is a capsular polysaccharide that is essential for the virulence of *S*. Typhi and has been shown to induce protective immunity in humans although by nature it is hypoimmunogenic and vaccines based on the Vi polysaccharide elicit a T-cell independent protection of a short duration^24^. Conjugation of Vi to a carrier protein has been used to substantially increase antibody response and efficacy in infants. We demonstrated that conjugation of Vi to CRM_197_ substantially increases the antibody response to Vi in mice and humans^25–27^ and no differences are observed by adjuvanting the Vi-CRM_197_ vaccine with Alhydrogel in mice^28^. In the Vi-CRM_197_, the source of Vi is from the less pathogenic *Citrobacter freundii* bacterium for enhanced safety in the operations of vaccine manufacture^28^. *C. freundii* Vi polysaccharide is structurally similar to, and immunologically indistinguishable from, the Vi polysaccharide of *S*. Typhi^29,30^. Additionally, we previously demonstrated that fragmentation of Vi to generate shorter-chain Vi conjugates induces in mice a purer T-dependent response in contrast to the retained T-independent response properties of long-chain Vi conjugates^26^.

The trivalent iNTS-TCV vaccine is currently undergoing clinical assessment. Specifically, it has recently completed a Phase 1/2a study in healthy adults in both non-endemic and endemic populations to evaluate its safety, tolerability, and immunogenicity [NCT05480800]^31^. Prior to the full trivalent formulation, the bivalent iNTS-GMMA component^32^, successfully completed a first-in-human Phase 1 trial in the United Kingdom, confirming the component’s safety and immunogenicity in healthy adults^32^. Following this, the iNTS-GMMA vaccine has advanced to a Phase 1 study in Kenya [PACTR202310834458532]^33^, and a Phase 2a age de-escalation trial in Ghana [NCT06213506]^34^, further progressing its evaluation in the target demographic of young children.

This approach of combining the iNTS-GMMA with a licensed TCV vaccine could be an effective strategy for the prevention of invasive salmonellosis caused by *S*. Typhimurium, *S*. Enteritidis and *S*. Typhi. However, there are also potential challenges associated with combining these two vaccine technologies. First, the immune response generated by a GMMA-based vaccine and the Vi-CRM_197_ vaccine may differ in terms of the types of immune cells and antibodies stimulated, with the potential for mutual interference that could adversely affect the overall immune response. Second, the combination of the two vaccines could lead to enhanced reactogenicity. Third, the matrix of the GMMA and Alhydrogel may give difficulties in generating a stable vaccine formulation in presence of the Vi-CRM_197_, thus making the vaccine manufacturability not feasible. In this study, we demonstrate the feasibility of combining the GMMA technology with the glycoconjugate vaccine platform in the iNTS-TCV vaccine candidate and the immune-enhancement effect that derives from the GMMA platform on the Vi-CRM_197_ vaccine in mice studies. Moreover, we describe the preclinical data generated to support clinical trials of the iNTS-TCV trivalent vaccine, including nonclinical immunogenicity and toxicology data of this candidate vaccine that supported progression to the Phase 1/2a clinical trial in healthy adults.

## Results

### The nontyphoidal Salmonella components of the iNTS-TCV vaccine candidate are based on the GMMA technology

The trivalent iNTS-TCV vaccine combines GMMA from *S. enterica* serovars Typhimurium (STmGMMA) and Enteritidis (SEnGMMA) with the Vi-CRM_197_ glycoconjugate of the licensed TCV TYPHIBEV (Biological E). The drug substances STmGMMA and SEnGMMA are suspensions of outer membrane vesicles produced from *S*. Typhimurium 2192 and *S*. Enteritidis 618 strains carrying knockout mutations in *tolR*, *msbB* and *pagP* to enhance vesicle release and reduce endotoxicity of lipid A^15,35^. GMMA are purified from fermentation broth and consist of cell-free bacterial proteins, lipids (including phospholipids), and OAg-containing lipopolysaccharide (LPS) suspended in NaCl. The production processes for STmGMMA and SEnGMMA have been published recently^16^. For the GMMA components of the iNTS-TCV vaccine, production used for the first time single-use bioreactor as a proof of concept for the scalability and suitability of this production technology for potential future use in LMICs. GMMA were produced by fermentation of the engineered production strains, which reached an OD_600_ of 45 ± 15 within 28 ± 5 h for *S*. Typhimurium and 12 ± 3 h for *S*. Enteritidis after bioreactor inoculation, coincident with a spike in dissolved oxygen (pO_2_) and a rise in pH. Cultures were harvested by microfiltration and GMMA were subsequently purified by ultrafiltration. GMMA from the two strains had similar homogeneous particle sizes: Z-average diameters were 114 nm for STmGMMA and 93 nm for SEnGMMA. OAg size was assessed by size-exclusion chromatography with refractive index detection after acetic acid extraction from GMMA; for both STmGMMA and SEnGMMA, two OAg peaks were observed: a medium molecular weight peak ranging from 32 to 36 kDa and a low molecular weight peak ranging from 1 to 3 KDa (corresponding to core only or core plus few OAg repeating units).

The STmGMMA and SEnGMMA batches were produced at 30-L scale to demonstrate manufacturability, process robustness and scalability. Their physicochemical characteristics, that are not dissimilar to previous batches produced in stainless-steel bioreactors^16^, are shown in Supplementary Table S1.

### Formulation and characterization of the iNTS-TCV vaccine

The iNTS-TCV vaccine formulation consists of SEnGMMA and STmGMMA, normalized by OAg content and adsorbed onto Alhydrogel, followed by addition of the Vi-CRM_197_ conjugate produced as previously described^36^. Only the GMMA components are adsorbed to the aluminum salt; the formulation was developed such that the majority of Vi-CRM_197_ (i.e. >60%, as quantified by HPAEC-PAD) remains in the supernatant. The target composition of the highest anticipated vaccine concentration in a 0.5 mL dose is: 40 μg OAg/mL of STmGMMA, 40 μg OAg/mL of SEnGMMA, and 50 μg Vi/mL of Vi-CRM_197_, formulated with 0.7 mg Al^3+^/mL Alhydrogel. Formulation conditions were developed at small scale with a simple, modular process, then scaled to 1 L to produce reference materials and a non-good manufacturing practices (GMP) toxicology lot at GVGH and GMP clinical materials at a Contract Manufacturing Organization (CMO). Formulations were characterized by a panel of analytical tests; Table 1 reports characterization data for multiple iNTS-TCV batches, confirming process robustness and comparability between non-GMP toxicology and GMP clinical materials.

The analytical panel was developed to characterize and control the quality attributes of both GMMA components and the Vi-CRM_197_ conjugate of this combination-platform vaccine. A Formulated-Alhydrogel competitive-ELISA (FAcE) assay, using OAg-specific monoclonal antibodies, quantified the individual OAg content from STmGMMA and SEnGMMA in formulation. For all lots, OAg values were 39–53 μg/mL for *S*. Typhimurium and 35–41 μg/mL for *S*. Enteritidis. Not adsorbed GMMA on Alhydrogel were determined by quantifying lipid A amount in the supernatant by HPLC-RP/MS and were confirmed to be low (≤ 10% of total material). Total and free Vi in the final drug product were quantified by HPAEC-PAD. Total Vi was measured directly on the Alhydrogel-formulated vaccine after an initial pretreatment by acid hydrolysis with TFA/HCl^37^; free (unconjugated) Vi was measured after precipitation of the glycoconjugate with deoxycholate (DOC), followed by centrifugation, and quantification of Vi in the drug product supernatant by HPAEC-PAD. Unconjugated Vi was reported as a percentage of total Vi. Total Vi was 43–47 μg/mL, and free (unconjugated) Vi was 5–9% of total for the different lots. The vaccine is supplied as a single-vial, whitish suspension, free of visible aggregates upon visual inspection, with osmolality 240–360 mOsm/kg and pH 6.0–7.0. Stability testing included real-time (5°C) and accelerated studies (25°C for 56 days). Formulation reference material and non-GMP toxicology lots produced at GVGH, and GMP clinical materials produced at the CMO, were assessed up to 42 months at real-time temperature, with key physicochemical attributes remaining within specification (Supplementary Table S2). Immunogenicity assessments in mice were performed as part of the real-time stability programme to assess vaccine potency by comparing a freshly prepared reference standard with the toxicology and GMP lots of iNTS-TCV stored at 2–8°C. Results from the 18-month stability time point potency study of the toxicology lot versus the freshly prepared reference standard are shown as representative data (S1 Fig.). A highly significant dose-response was observed for each component after both one and two immunizations on days 27 and 42, respectively (Spearman’s rank correlation, P < 0.001 for all components). Linear regression of log-transformed ELISA units versus log-transformed doses yielded similar slopes and Y-intercepts for reference standards and toxicology lots for each component. Together, these data indicate no detectable loss of potency after 18 months of storage at 2–8 C (S2 Fig.).

### Immunogenicity of the iNTS-TCV vaccine candidate in animal studies

The potential for immunologic interference between the two GMMA components and the Vi-CRM_197_ conjugate was evaluated in mice. Animals were immunized with STmGMMA/Alhydrogel, SEnGMMA/Alhydrogel, Vi-CRM_197_ alone, or the combined iNTS-TCV vaccine formulation across a 4-fold dose range. Both GMMA components elicited high, dose-dependent levels of specific anti-*S*. Typhimurium OAg and anti-*S*. Enteritidis OAg serum IgG. ELISA titers for each OAg did not differ when the antigen was administered alone or as part of the iNTS-TCV formulation (Fig. 1A, B). Antisera showed strong bactericidal activity against homologous *S. enterica* strains in serum bactericidal assays (SBA) (Fig. 1, D, E), and no negative immunologic interference was observed by either ELISA or SBA. Unexpectedly, combining TCV with GMMA components increased the anti-Vi IgG response: anti-Vi IgG titers (Fig. 1C) and SBA activity against *C. freundii* (Fig. 1F) induced by the iNTS-TCV formulation were significantly higher than those induced by TCV alone containing an equivalent amount of Vi-CRM_197_.

We assessed IgG subclass distribution to characterize the quality of the immune response elicited by the combination-platform iNTS-TCV compared with the single components. GMMA, whether administered alone or as part of iNTS-TCV, induced anti-*S*. Typhimurium OAg (Fig. 2A) IgG responses with comparable proportions of IgG1, IgG2a, IgG2b and IgG3; although for the anti-*S*. Enteritidis OAg (Fig. 2B) the anti IgG response elicited by the iNTS-TCV showed a higher proportion of IgG3 compared to the single GMMA component. By contrast, Vi-CRM_197_ induced an anti-Vi response dominated by IgG1 with only minor IgG2a, IgG2b and IgG3. The iNTS-TCV elicited a different subclass profile against Vi, with substantially higher proportions of IgG2b and IgG3 compared with the response induced by Vi-CRM_197_, in which these subclasses were almost absent (Fig. 2C).

### Intramuscular rabbit pyrogen test (mRPT) and monocyte activation test (MAT) to assess relative pyrogenicity of the iNTS-TCV vaccine candidate

We investigated the preclinical tolerability of the iNTS-TCV vaccine by performing a modified intramuscular rabbit pyrogen test (mRPT) and a Monocyte Activation Test (MAT). The mRPT was conducted with the iNTS-TCV formulation administered to the highest anticipated human dose and injection volume (20 μg OAg of each GMMA and 25 μg Vi polysaccharide per 0.5 mL dose). The iNTS-GMMA vaccine (i.e., the mixture of STmGMMA/Alhydrogel and SEnGMMA/Alhydrogel, 20 μg OAg of each GMMA per 0.5 mL dose) was included as control. Maximal temperatures of the individual rabbits did not exceed 41°C and returned to baseline within 24 hours; the observed fever responses were transient, of acceptable magnitude, and overall tolerability was good (Supplementary Table S3).

A MAT (Ph. Eur. Chapter 2.6.30 Method 2) was developed to characterize iNTS-TCV and as potential replacement for the rabbit test. GMP-produced GMMA from *S*. Typhimurium and *S*. Enteritidis production strains tested in the pyrogenicity study were used as comparator to calculate the relative pyrogenic unit (RPU) of iNTS-TCV. The MAT was introduced into the lot-release/characterization panel for clinical batches in place of the mRPT. RPU values, calculated relative to reference lots, were close to 1 (Table 1).

### In vitro immuno-profiling to dissect the GMMA-driven cytokine release pattern from human peripheral blood mononuclear cells (PBMCs)

We further investigated how the GMMA component contributes to the enhanced anti-Vi IgG response and functionality of the iNTS-TCV vaccine. Because GMMA carry abundant pathogen-associated molecular patterns (PAMPs) that can recruit and activate polymorphonuclear cells (PMNs, e.g., neutrophils) to produce cytokines linked to inflammation (e.g., IL-6, TNF-α, IL-1β) and chemokines linked to immune stimulation^38^, and because cytokines can influence human IgG subclass distribution^39^, we performed an in-depth *in vitro* cytokine profiling. Human PBMCs were exposed to iNTS-TCV, the STmGMMA and the SEnGMMA components, and the Vi-CRM_197_ glycoconjugate (TCV) alone. GMMA were the main drivers of cytokine and chemokines secretion from PBMCs (Fig. 3); inclusion of the Vi-CRM_197_ glycoconjugate (TCV) and/or Alhydrogel did not substantially alter the cytokine pattern compared with the mix of STmGMMA and SEnGMMA drug substances lacking Vi-CRM_197_ and Alhydrogel. While numerous chemokines and cytokines involved in immune modulation were induced in the presence of GMMA components, the Vi-CRM_197_ alone elicited less chemokines and cytokines induction at the concentration tested.

### Nonclinical Safety profile of the iNTS-TCV vaccine candidate

A Good Laboratory Practice (GLP) repeated-dose toxicology study was performed in New Zealand White (NZW) rabbits to assess local tolerance and potential systemic toxicity of the iNTS-TCV vaccine. Rabbits received four intramuscular administrations of iNTS-TCV (20 μg in OAg of STmGMMA, 20 μg in OAg of SEnGMMA, 25 μg in Vi of Vi-CRM_197_ per 0.5 mL dose) at 2-week intervals. A NZW rabbit control group received saline at the same immunization schedule. Necropsies were performed on half the animals 3 days after the last dose (main group) and on the remaining animals 28 days later (recovery group). The regimen was well tolerated: there were no vaccine-related deaths during the study, no ophthalmological findings, and no treatment-related changes in body weight or food consumption. Overall, the vaccine-related changes were of low severity and likely represented a physiological reaction following the immune stimulation in response to the aluminum-containing vaccine candidate and there was no evidence of systemic toxicity. Local reactions to vaccine injections consisted mainly of edema and induration of higher grade in iNTS-TCV-treated animals, in comparison to the control group. These reactions generally resolved within 2 weeks or decreased in severity over time and were considered non-adverse and consistent with the expected inflammatory and immune response following vaccine administration. An increase in mean body temperature was observed for iNTS-TCV-treated animals, 2 hours post-dose, after the first 3 administrations, and 6 hours post-dose following the last one, and returned close to baseline values within 24 hours. In view of the short duration and low amplitude of this increase, this was not considered as toxicologically relevant. Compared to control values, slight changes in white blood cells (heterophils, large unstained cells and monocytes), coagulation (prothrombin time, activated partial thromboplastin time and fibrinogen) and serum clinical chemistry parameters (cholesterol, triglycerides, phospholipids and globulin), as well as an increase inC-reactive protein (CRP) levels were noted in iNTS-TCV-treated animals. These reversible effects were consistent with signs of inflammatory changes observed microscopically at injection sites and were considered to reflect immune activation.

At terminal euthanasia (Day 46), i.e., 3 days after the fourth injection, gross lesions associated with vaccine administration at the injection sites consisted of dark or pale foci, histopathologically characterized by subacute/chronic mixed inflammation, macrophages aggregation, amphophilic foreign material accumulation (inflammatory cell debris and vaccine compound) that correlated with inflammatory changes. The increases in lymphoid cellularity in the lymph nodes (iliac, inguinal, sacral and popliteal), noted in most treated males and females compared with controls, were considered to be secondary to the stimulation of the immune system by the vaccine. More in detail these findings correlated microscopically with inflammatory changes in the subcutaneous tissue and/or muscle layer, characterized by subacute inflammation, macrophage aggregation, and subcutaneous fibroplasia. After the 4-week treatment-free period, at the recovery sacrifice on Day 71, the changes noted at the injection sites and in the lymph nodes were partially resolved, indicating that the post-vaccinal recovery was ongoing.

In the non-GLP immunogenicity phase of the study, iNTS-TCV injected intramuscularly, was highly immunogenic. Increased anti-*S*. Typhimurium OAg, anti-*S*. Enteritidis OAg, and anti-Vi serum IgG ELISA units were noted on Days 15, 29, 43, 46 and 71 compared with pre-treatment and concurrent control animal values, indicating a specific antibody response (Fig. 4 A–C). In addition, strong homologous bactericidal activity against the vaccine strains was noted on Days 15 and 46 for the iNTS-TCV-treated animals compared to control animals (Fig. 4 D–F).

## Discussion

No vaccines are currently licensed to prevent iNTS disease, which primarily affects children under five years of age in sSA and is a major public-health concern. Several candidates are in development; the most advanced combine NTS antigens with licensed TCVs to produce trivalent formulations targeting *S*. Typhimurium, *S*. Enteritidis, and *S*. Typhi. These trivalent vaccines could reduce the approximately 155,000 deaths caused by iNTS disease and typhoid fever annually^3,9^. To date, one of most advanced *Salmonella* combination vaccines targeting S. Typhimurium/*S*. Enteritidis/*S*. Typhi is being developed in partnership between the University of Maryland and Bharat Biotech International Ltd^40^. The vaccine includes two NTS glycoconjugates, containing core-O-polysaccharides of *S*. Typhimurium and *S*. Enteritidis coupled to their respective flagellin antigens, in combination with the licensed Typbar-TCV (a glycoconjugate of Vi linked to tetanus toxoid). This vaccine has been tested in Phase 1 and Phase 1/2a studies in healthy adults in the United States and found to be safe and immunogenic with long-lasting antibody responses^41^. These results supported progression to a Phase 2 age-descending study in sSA (NCT05784701).

The GVGH iNTS-TCV vaccine is the first combination-platform vaccine that combines GMMA technology with glycoconjugates. The vaccine contains GMMA from *S*. Typhimurium and *S*. Enteritidis together with the TYPHIBEV TCV, a Vi-CRM_197_ glycoconjugate licensed by Biological E Ltd. The GMMA technology enables simple, robust and high-yield vaccine production. Additionally, in this work we have used, for the first time, single-use bioreactor to generate GMMA, which could be particularly suited for LMICs. Single-use bioreactors maintain traditional stirred-tank fermentation design principles, performance and scalability, but have the advantage of being easily implemented in GMP facilities, limiting operational costs (e.g., cleaning and its validation), enabling easier and faster change-over, and ultimately accelerating clinical manufacturing.

The iNTS-TCV vaccine, which combines GMMA and glycoconjugates, was found to be highly immunogenic in both mice and rabbits; low doses (in the ng range of OAg and Vi) induced high levels of anti-*S*. Typhimurium OAg, anti-*S*. Enteritidis OAg and anti-Vi antibodies. As one of the challenges of combination vaccines is potential immunological interference, we evaluated the immune response to the iNTS-TCV vaccine in mice in comparison with its individual components. No substantial differences were observed in serum anti-*S*. Typhimurium OAg and anti-*S*. Enteritidis OAg IgG responses or in antibody functionality against *S*. Typhimurium and *S*. Enteritidis when comparing the individual vaccine components STmGMMA/Alhydrogel and SEnGMMA/Alhydrogel with their responses when included in the iNTS-TCV vaccine. In addition, anti-Vi IgG titers and SBA activity against *C. freundii* elicited by the iNTS-TCV vaccine were significantly higher than those elicited by the individual Vi-CRM_197_ component at the same dose. If observed also in human clinical trials, this could represent a powerful strategy to achieve dose sparing for TCV.

The presence of GMMA in the iNTS-TCV vaccine candidate not only enhanced the anti-Vi response in mice but also altered the type of immune response elicited against Vi. IgG subclass analyses showed that anti-Vi IgG2b and IgG3 were induced by the iNTS-TCV vaccine at higher levels than by Vi-CRM_197_ alone. This difference in subclass profile, driven by the GMMA components, may contribute to the enhanced antibody functionality observed. The immunoprofiling data obtained in this work supports this hypothesis: the GMMA components within iNTS-TCV were the primary drivers of cytokine secretion from human PBMCs, inducing a robust release of chemokines and cytokines that are key modulators of the immune response and antibody production. The iNTS-TCV vaccine elicited same chemokine and cytokine induction pattern as PBMCs exposed to an equal mixture of the STmGMMA and SEnGMMA components, whereas Vi-CRM_197_ alone elicited lower chemokine and cytokine induction. In particular, in addition to the inflammatory cytokines IL-6, IL-1β and the TNF-α, which drive acute-phase responses and were completely absent in Vi-CRM_197_-stimulated PBMCs, IL-8 (a potent chemoattractant that rapidly recruits neutrophils to the site of inflammation), MCP-1 (which recruits monocytes and dendritic cells to sites of tissue injury), and MIP-1α (which recruits and activates monocytes, B cells, and T cells) were primarily induced by the GMMA component of the iNTS-TCV vaccine^42–45^. This suggests that the isotype switching of B cells observed in mice, leading to increased IgG2b and IgG3, which are subclasses associated with enhanced complement activation and opsonophagocytic activity, may be linked to the GMMA-induced cytokine milieu. Indeed, the localised production of TNF-α and IL-6, IL-8, MCP-1, and MIP-1α can create a dense, localized inflammatory hub at the injection site. In fact, as demonstrated by histopathological analysis in the repeated-dose toxicology study, this environment results in the recruitment of macrophages, inflammatory dendritic cells, and lymphocytes to the injection site and as well in increased lymphoid cellularity in the draining lymph nodes. This immunostimulatory effect of GMMA could explain the overall increase in Vi-specific antibody titers and bactericidal activity. Indeed, the importance of multiple immunoglobulin subclasses in limiting dissemination of *Salmonella* infection and conferring protection has been demonstrated^46–48^. Taken together, these data support the hypothesis that GMMA in the formulation can act as an adjuvant in its own. In fact, Alhydrogel has been used in preclinical and clinical studies with GMMA vaccine formulation as an adsorbent agent, with the main purpose of reducing potential GMMA systemic reactogenicity, and not as an adjuvant^49^. The observed adjuvant effects of GMMA within the iNTS-TCV formulation may not only enhance the magnitude of the Vi-specific antibody response, but also extend the duration of immune protection against typhoid fever; this hypothesis will be tested in upcoming clinical trials.

Despite the fact that immune mechanisms of protection against typhoid fever can only be fully evaluated in humans, due to the restriction of the pathogen to human hosts, the results in mice highlight the possibility that the iNTS-TCV vaccine could stimulate different IgG subclasses, and with different magnitude, in humans compared with Vi-CRM_197_-based vaccines.

The strong immunogenicity observed in mice facilitated the development of a potency test that compares the relative immunogenicity of the iNTS-TCV vaccine to a freshly formulated standard. This potency assay, together with biochemical stability assessments, has allowed assignment of a shelf-life of up to 42 months of storage at 5°C based on current data, demonstrating that generation of a stable formulation of this combination-platform vaccine through a simple manufacturing process is feasible. To support the preclinical safety of the iNTS-TCV vaccine and demonstrate that combining GMMA components with Vi-CRM_197_ does not increase reactogenicity, a repeated-dose toxicology study in rabbits and MAT assays (replacing the modified rabbit pyrogen test) on the final drug product batches were conducted. Use of the MAT overcomes species-dependent differences in TLR specificity, which may affect the translatability of results obtained in rabbits to humans^50,51^. Both the *in vitro* and *in vivo* analyses indicate an acceptable safety profile of this vaccine.

The manufacturability of iNTS-TCV, its favourable stability profile, and the serum IgG and functional antibody responses elicited by the vaccine components, together with the preclinical safety data, supported progression to a Phase 1/2a study [NCT05480800]^31^. Based on this data package, the trivalent iNTS-TCV vaccine candidate, which combines iNTS-GMMA with TCV, has the potential to provide an effective, affordable and sustainable vaccine for populations most at risk, offering protection against both iNTS disease and typhoid fever and contributing to reduced antibiotic use in sSA.

## Materials and Methods

### Generation of S. Typhimurium and S. Enteritidis GMMA-producing strains

*Salmonella* Typhimurium wild-type (WT) strain 2192^52,53^ and *Salmonella* Enteritidis WT strain 618^53,54^ were chosen as parent strains. The vaccine production strains *S*. Typhimurium Δ*tolR*::*aph* Δ*msbB::tetRA* Δ*pagP*::*cat* and *S*. Enteritidis Δ*tolR*::*aph* Δ*msbB::tetRA* Δ*pagP*::*cat* were obtained as previously described^55 15^. Briefly, the *tolR* gene was replaced with the kanamycin resistance gene *aph*, the *msbB* gene was replaced with the tetracycline resistance genes *tetRA* and the *pagP* was replaced with the chloramphenicol resistance gene *cat* using the same strategy and primers described by Rossi *et al*^56,55,15^. Master Cell Banks (MCB) were produced by a CMO under GMP conditions as previously reported^16^.

### Production and purification of STmGMMA and SEnGMMA using single-use bioreactors

Fermentation and GMMA purification were adapted from previously published methods^16^ and modified for single-use bioreactor (HyPerforma Enhanced Single-Use Fermentor, e.S.U.F.; Thermo Fisher Scientific). For production lots, a flask with 600 mL of defined medium (DM^16,35^) was inoculated with a culture from MCB and incubated at 30°C with agitation (200 rpm). Seed cultures were started at OD_600_ of 0.2–0.6 and grown to OD_600_ 4.5 ± 2.0, then used to inoculate the single-use bioreactor at 2% (v/v). Fermentation was controlled at pH 6.7 (adjusted with 25–28% NH_4_OH), 30°C, dissolved oxygen at 30% (controlled), airflow 15–30 standard liters per minute (SLPM) and stir speed 50–800 rpm (cascade) until a final OD_600_ of 45 ± 15 was reached. Process parameters were optimized for single-use operation by introducing an O_2_ flow cascade because in single-use bioreactors pressure cannot be used as a cascade variable for dissolved oxygen (DO) control due to the structural and operational limitations of the system; therefore, control is achieved by modulating the oxygen flow, which ensures greater process safety, responsiveness, and stability.

GMMA were purified using the method described previously^16^. Briefly, GMMA released into the fermentation broth were recovered by two consecutive tangential flow filtration (TFF) steps: microfiltration to separate culture supernatant (containing GMMA) from the bacterial cells, and ultrafiltration to concentrate GMMA and remove soluble proteins and nucleic acids.

Characterization for drug substance lot release included: total OAg content by HPAEC-PAD; total protein by micro-BCA; lipid A quantification by HPLC-RP MS; particle size by dynamic light scattering; soluble protein by quantification of protein in the supernatant after GMMA ultracentrifugation; O-acetyl content by NMR or Hestrin colorimetric assay; OAg molecular size distribution by HPLC-SEC with semicarbazide derivatization; and lipid A structure by MALDI-TOF, as previously described^57,58^.

### Formulation of iNTS-TCV vaccine candidate

The Vi-CRM_197_ drug substance was supplied by Biological E Limited (Hyderabad, India). For iNTS-TCV drug product formulation at 1 L scale, sterile-filtered suspensions (Sartobran P150) of STmGMMA (starting buffer: 70 mM NaCl) and SEnGMMA (starting buffer: 55 mM NaCl), each at 1000 μg/mL OAg, were prepared. The combined GMMA suspension was added to aluminium hydroxide (Alhydrogel 2% wet gel suspension; Brenntag) in water and stirred for 2 hours at room temperature (180 rpm), yielding an initial OAg concentration of 80 μg/mL. Sodium phosphate was then added to a final concentration of 20 mM while mixed (180 rpm) for 30 minutes, and subsequently osmolarity was adjusted with 1.54 M NaCl to achieve 154 mM NaCl; the formulation was mixed for a further 15 minutes at 180 rpm. Vi-CRM_197_ was added with continuous mixing to a final Vi content of 50 ug/mL Vi and the suspension was mixed for 30 min at room temperature (180 rpm). The bulk was filled at 0.7 mL per 3 mL glass vial and stored at 2–8°C.

The final formulated product contained 40 μg OAg/mL STmGMMA, 40 μg OAg/mL SEnGMMA, 50 μg Vi/mL (Vi-CRM_197_), and 0.7 mg Al^3+^/mL in 20 mM phosphate, 154 mM NaCl, pH 6.5, 300 mOsm/kg. The formulation was tested for identity, OAg and Vi content, total protein, aluminum content, extractable volume, non-adsorbed protein, adsorbed Vi, visual appearance, pH, osmolality, sterility, and immunogenicity and was placed on stability studies. GVGH consistency runs produced reference standards and non-GMP toxicology lots, and a CMO manufactured the GMP Phase 1/2a clinical lots. A GMP placebo diluent (Alhydrogel 0.7 mg Al^3+^/mL, 20 mM phosphate, 154 mM NaCl) was prepared and tested for identity, aluminum content, extractable volume, visual appearance, pH and osmolality. The iNTS-TCV and placebo GMP lots met the defined specifications and were used in a Phase 1/2a study in healthy adults in both non-endemic and endemic populations [NCT05480800]^31^. Manufacturing of the iNTS-TCV vaccine and the placebo batches was performed in compliance with GMP guidelines for manufacture of sterile medicinal products and investigational medicinal products in adequate GMP facilities. To ensure product sterility, environmental monitoring, media-fill simulations and final sterility testing of the formulated bulk and filled products at release were performed.

Single GMMA component formulations (STmGMMA/Alhydrogel and SEnGMMA/Alhydrogel) were prepared by adding a suspension of either STmGMMA or SEnGMMA (80 μg/mL OAg in 154 mM NaCl) to Alhydrogel in water to achieve a final OAg concentration of 40 μg/mL, stirring (90 rpm) for 2 hours at room temperature, and then adjusting by addition of NaCl and phosphate to obtain the desired osmolarity (240–360 mOsm/kg) and pH (5.5–7.5).

### Identity and quantification of active ingredients in the iNTS-TCV drug product and measurement of total and not adsorbed protein to Alhydrogel

STmGMMA and SEnGMMA OAg were quantified by HPAEC-PAD directly on the Alhydrogel formulation as previously described^58^. Individual OAg components in the final drug product were quantified by FacE, which specifically detects *S*. Enteritidis and *S*. Typhimurium OAg polysaccharides in formulated samples^59^. Identity and quantification of Vi polysaccharide in the final drug product were performed by HPAEC-PAD after acid hydrolysis of the sample: Vi is hydrolysed to the monomer of the repeating unit, which corresponds to the target chromatographic peak as described previously^37^. For determination of unconjugated (free) Vi, the glycoconjugate in the vaccine drug product was first precipitated with deoxycholate (DOC) and the supernatant was then analysed by HPAEC-PAD. After hydrolysis, Vi quantification is performed by HPAEC-PAD analysis with a Dionex ICS3000 (or 5000) on a CarboPac PA1 column coupled with PA1 guard column. Separation is performed at 25°C using the following condition: 15 min, 400 mM NaOH, flow rate 1.5 mL/min; and quantification of Vi content in the drug substance and the drug product samples is done using a Vi calibration curve in the range 0.16–5 μg/mL.

### Measurement of GMMA and Vi polysaccharide not adsorbed to Alhydrogel, in the iNTS-TCV final formulation

GMMA not adsorbed to Alhydrogel was quantified as lipid A in drug product supernatant obtained after 0.2 μm centrifugal filtration (Nanosep); lipid A was measured by HPLC-RP/MS as previously described^58^. Vi not adsorbed to Alhydrogel was quantified in drug product supernatant after 0.2 μm centrifugal filtration by Nanosep of the vaccine sample. Main data to show consistency of the process are presented for one lot produced at GVGH (reference iNTS-TCV formulation) and one lot produced under GMP conditions in a CMO.

### Modified pyrogenicity test in rabbits

For the assessment of GMMA-induced fever response in rabbits, a modification of the European Pharmacopeia pyrogenicity test method (Ph. Eur. 2.6.8 pyrogens^60^) was used. This modification allows intramuscular administration of the full human dose, as previously described^35^. Briefly, rabbits divided into groups of three were placed in retaining boxes and body temperatures were recorded using a rectal probe. The iNTS-TCV (20 μg OAg *S*. Typhimurium GMMA, 20 μg OAg *S*. Enteritidis and 25 μg Vi polysaccharide per dose) and the iNTS-GMMA vaccine as comparator (20 μg OAg *S*. Typhimurium GMMA, 20 μg OAg *S*. Enteritidis per dose) were administered intramuscularly in 0.5 mL. Sterile physiological saline was injected into the three rabbits of the control group serving primarily as the negative control and ensured that any temperature observed in the rabbits was due to the test substance, not to the vehicle or to the handling process. Temperatures were recorded as recently described^16^.

### Monocyte Activation Test (MAT) on drug product and in vitro immuno-profile

MAT on the formulated iNTS-TCV vaccine was performed following Ph. Eur. Chapter 2.6.30 Method 2. The *in vitro* interleukin 6 (IL-6) production by human PBMCs following stimulation with the STmGMMA and SEnGMMA drug substances mixture as reference and the iNTS-TCV vaccine as test was evaluated using a setup similar to that described previously for GMMA-based vaccine^50^.

For the *in vitro* immunoprofiling, cytokine and chemokine expression was assessed using the LEGENDplex Human Proinflammatory Chemokine Panel 1 (12-plex) with V-bottom plate (cat. No. 741081) and the Human Inflammation Panel 1 (13-plex) with V-bottom plate (cat. No. 740809), following the manufacturer’s instructions. Briefly, supernatants from PBMCs stimulated with the STmGMMA and SEnGMMA drug substances mixture and the iNTS-TCV vaccine were incubated for 2 h with beads pre-coated with specific antibodies. Beads are divided into two groups based on their size (A and B) and each bead has a specific internal fluorescence. Cytokines and chemokines are captured by bead-bound antibodies, then incubated for 1 h with a biotinylated detection antibody, followed by a 30-min incubation with streptavidin-phycoerythrin (SA-PE). Because the beads are distinguished by size and internal fluorescence intensity on a flow cytometer, analyte-specific bead populations can be segregated and the PE fluorescent signal quantified using the manufacturer’s analysis software.

### Immunogenicity in mice

To assess the immunogenicity of the iNTS-TCV vaccine, eight female CD1 mice (4 to 6 weeks old) per group received intraperitoneal injections of different OAg doses of STmGMMA/Alhydrogel or SEnGMMA/Alhydrogel, or Vi-CRM_197_ or the iNTS-TCV vaccine in a volume of 0.5 mL. Doses ranged between 0.01 μg and 0.63 μg of each GMMA OAg (based on 4-fold dilutions) and between 0.012 μg and 0.78 μg of Vi polysaccharide. Mice received two immunizations on Days 0 and 28, and blood samples were collected on Days 27 and 42. No placebo group was included for reduction purposes and post-vaccination titers were compared with pre-immune titers.

In immunogenicity studies, the responses elicited by a range of doses of STmGMMA/Alhydrogel or SEnGMMA/Alhydrogel and Vi-CRM_197_ administered as single components were compared with the immunogenicity of the iNTS-TCV vaccine containing the same amounts of STmGMMA, SEnGMMA and the Vi-CRM_197_ components. ELISA analyses were performed to determine levels of anti-*S*. Typhimurium OAg, anti-*S*. Enteritidis OAg, and anti-Vi polysaccharide IgG antibodies using a methodology similar to that previously applied for *S*. Typhimurium and *S*. Enteritidis OAg quantification^61^ and Vi quantification^62^, as detailed in the section ‘Enzyme-linked immunosorbent assay (ELISA)’. SBA, as detailed in section ‘Luminescence-Serum bactericidal assay (L-SBA)’, was assessed to evaluate the functional activity of antibodies against *S*. Typhimurium strain D23580, *S*. Enteritidis strain CMCC4314 and *C. freundii* Vi-expressing strain 3056, as previously described^21,63,64^. *S*. Typhimurium D23580 was isolated from a child in Malawi with bloodstream infection^65^ and *S*. Enteritidis CMCC4314 is a laboratory strain^66^. Immunogenicity studies in mice were also used as a measure of vaccine component potency, using a similar setup comparing groups of mice immunized with four different OAg doses of the iNTS-TCV vaccine (toxicology or GMP lots) at different time points after production with a freshly formulated iNTS-TCV potency standard. The relative potency of vaccine and potency standard was determined by the estimated difference in Y-intercept divided by the estimated slope, as previously described^35^, using Combistats software.

### Toxicity study in rabbits

The study was conducted in New Zealand White rabbits (male and female, > 1.5 kg) in compliance with GLP standards at Charles River. Rabbits were assigned to two groups: one received the highest anticipated human dose of the iNTS-TCV vaccine (20 μg in OAg content from STmGMMA, 20 μg in OAg content from SEnGMMA, 25 μg Vi of Vi-CRM_197_ and 0.7 mg/mL Al^3+^ in 20 mM phosphate, 154 mM NaCl, pH 6.5, in a final volume of 0.5 mL), and the control group received 0.9% saline. The vaccine was administered four times, at two-weeks intervals, by the intramuscular route, followed by a four-week recovery period. All animals were monitored throughout the study for morbidity/mortality, clinical observations/examination, Draize injection site reactions, ophthalmology, body weights, and food consumption. Clinical pathology, macroscopic observations at necropsy, organs weights, and histopathology (complete WHO tissue list) were also assessed. Body temperature was recorded after each immunization. A non-GLP immunogenicity phase of the study was also performed.

### Immunogenicity in rabbits as part of the non-GLP immunogenicity phase of the toxicology study

Immunogenicity was assessed as part of the non-GLP immunogenicity phase of the GLP iNTS-TCV vaccine toxicology study by measuring serum IgG responses against *S*. Typhimurium OAg, *S*. Enteritidis OAg, and Vi polysaccharide in rabbits immunized with iNTS-TCV or placebo on Days 15, 29, 43, and 71.

### Enzyme-linked immunosorbent assay (ELISA)

IgG antibodies elicited against OAg from *S*. Typhimurium and *S*. Enteritidis, and against Vi polysaccharide, following immunization with single-component STmGMMA, SEnGMMA or Vi-CRM_197_ formulations, or with the trivalent iNTS-TCV vaccine, were assessed by ELISA. Plates were coated with OAg/polysaccharide (*S*. Typhimurium and *S*. Enteritidis OAg, or Vi polysaccharide), blocked with 5% milk in PBS, and incubated with sera diluted 1:100, 1:4,000 and 1:160,000 in PBS-0.05% Tween-0.1% BSA (mouse sera) or 5%milk in PBS (rabbit sera). Bound antibodies were detected using enzyme-labelled secondary antibodies (anti-mouse IgG, IgG1, IgG2, IgG3, IgG4- alkaline phosphatase or anti-rabbit IgG-alkaline phosphatase) in PBS-0.05% Tween-0.1% BSA. Immunoreactive anti-OAg /polysaccharide IgG was revealed by addition of substrate solution and measurement of absorbance at 405 nm, corrected by subtraction of absorbance at 490 nm. Samples were tested in comparison with calibrated mouse or rabbit antigen-specific reference sera, and results were expressed as ELISA units/mL relative to the reference serum. One ELISA unit was defined as the reciprocal of the dilution of the reference serum that yielded an OD of 1 in the assay. For IgG subclasses analysis, for which a reference serum was not available, a theoretical curve has been used, with starting ELISA units of 100 and 3 dilution fold steps.

### Luminescence-serum bactericidal assay (L-SBA)

Bactericidal activity of individual rabbit sera (Days 15 and 46) or mouse sera (Day 42) was evaluated against *S*. Typhimurium strain D23580, *S*. Enteritidis strain CMCC4314^66^ and *C. freundii* strain 3056, a Vi-expressing laboratory strain, using an L-SBA as previously described^63^. Assay results were expressed as IC50, defined as the reciprocal serum dilution resulting in a 50% reduction in luminescence, corresponding to 50% inhibition of bacterial growth. GraphPad Prism 7 software was used for curve fitting and IC50 determination. For samples with titers below the minimum measurable signal, a value equal to half of the first dilution tested was assigned.

## Supplementary Material

Supplementary Files

This is a list of supplementary files associated with this preprint. Click to download.

- Supportinginformation.docx

## Figures and Tables

**Figure 1:**
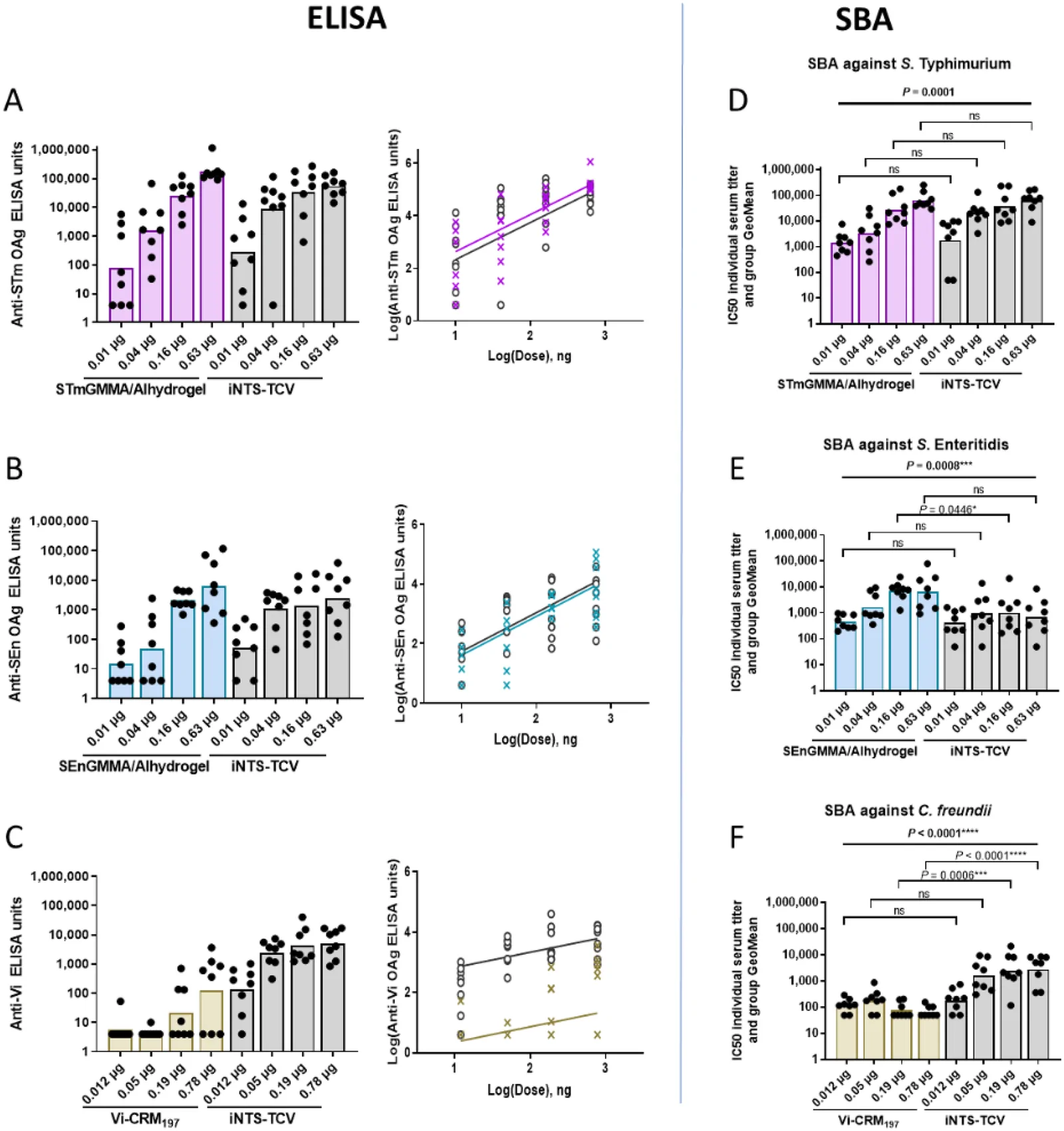
Comparison of the specific IgG levels and SBA activity of antisera from mice immunized with the individual STmGMMA/Alhydrogel, SEnGMMA/Alhydrogel and Vi-CRM_197_ or the iNTS-TCV formulation. **(A–C) ELISA**: Graph showing antigen-specific IgG distribution in mice immunized with STmGMMA or SEnGMMA formulated on Alhydrogel, or Vi-CRM_197,_ administered either as single components or as part of the iNTS-TCV formulation. Sera were assessed by ELISA at bleed day 42. Purified OAg from *S*. Typhimurium (A) or *S*. Enteritidis (B), and Vi polysaccharide (C) were used as coating antigens. Black circles indicate antigen-specific IgG detected from individual mice; bars represent group geometric means. Parallel-line analysis (PLA) of the log-transformed dose-response curves showed no significant differences in slopes and Y-intercepts between the individual components and iNTS-TCV for anti-STm OAg (P = 0.8166) and anti-SEn OAg (P = 0.1491). For the anti-Vi response, the Y-intercepts were significantly different (P = 0.0003), indicating a stronger response elicited by iNTS-TCV than the single Vi component. STm: *Salmonella enterica* serovar Typhimurium; SEn: *Salmonella enterica* serovar Enteritidis; OAg: O-antigen. **(D–F) SBA:** Serum bactericidal assay (SBA) IC50 values measured at bleed day 42 from mice immunized with STmGMMA or SEnGMMA formulated on Alhydrogel, or with Vi-CRM_197_, administered as single components or combined in the iNTS-TCV formulation. Homologous bactericidal responses against *S*. Typhimurium D23580 (D) and *S*. Enteritidis CMCC4314 (E) induced by the individual components were not significantly different from the responses elicited by the iNTS-TCV formulation. Bactericidal activity of Vi-CRM_197_ against *C. freundii* 3056 (F) was lower than the induced by the iNTS-TCV formulation. GMMA OAg concentrations in the formulations ranged from 0.01 μg to 0.63 μg OAg per dose; Vi polysaccharide concentrations ranged from 0.012 μg to 0.78 μg per dose. Single-component groups are shown in purple (STmGMMA/Alhydrogel), blue (SEnGMMA/Alhydrogel), and gold (Vi-CRM_197_); iNTS-TCV groups are shown in grey. Black circles indicate IC50 values from individual mice; bars represent group geometric means. Horizontal lines indicate the comparisons performed (single component versus the corresponding dose in iNTS-TCV). Statistical analysis was performed using the Kruskal Wallis test (as there was not always a significant dose-response and thus PLA could not be applied); when significant, pairwise comparisons were made with Dunn’s test with Bonferroni correction. Asterisks denote statistically significant differences; ns = not significant.

**Figure 2:**
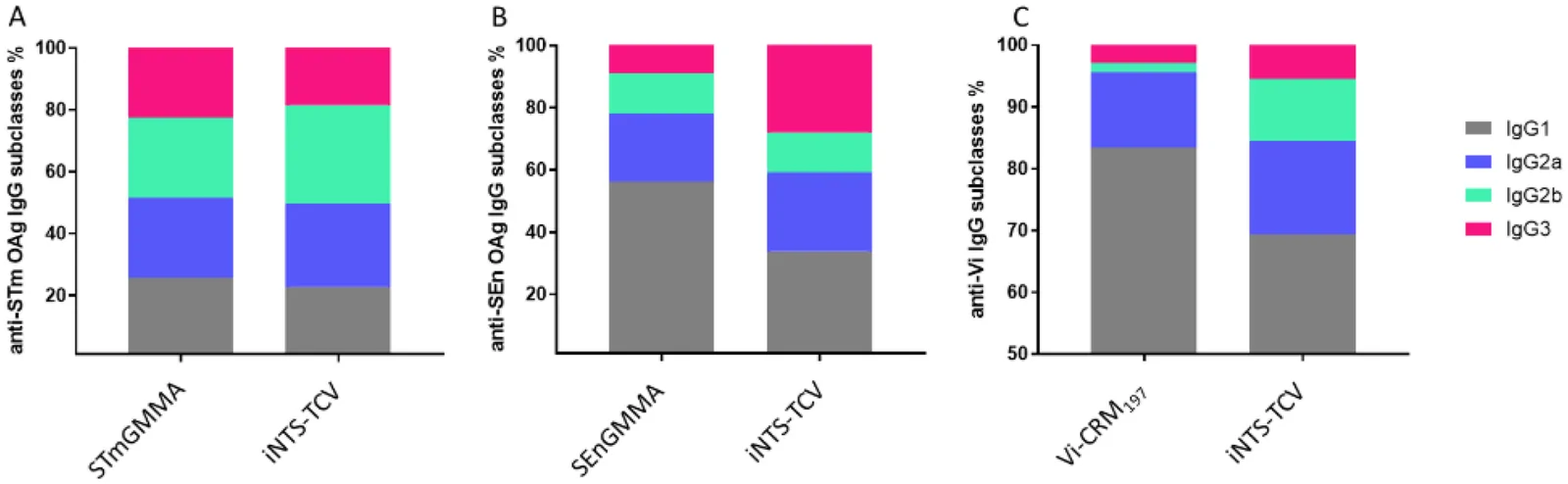
IgG subclass analysis. Graphs showing antigen-specific IgG subclass distribution (%) in mice immunized with STmGMMA or SEnGMMA formulated on Alhydrogel, or Vi-CRM_197_, administered either as single components or as part of the iNTS-TCV. Vaccine components were given at OAg concentrations of 0.63 μg per dose for STmGMMA or SEnGMMA and 0.78 μg per dose for Vi; sera were assessed by subclass-specific ELISA on day 42. OAg purified from *S*. Typhimurium (A) or *S*. Enteritidis (B), and Vi polysaccharide (C) were used as coating antigens. Antigen-specific IgG subclass distribution (%) was calculated for each mouse as the ratio between the EU/mL for the subclass of interest and the sum of titers for all IgG subclasses for that mouse, multiplied by 100; for each group, data are presented as the mean of the results.

**Figure 3:**
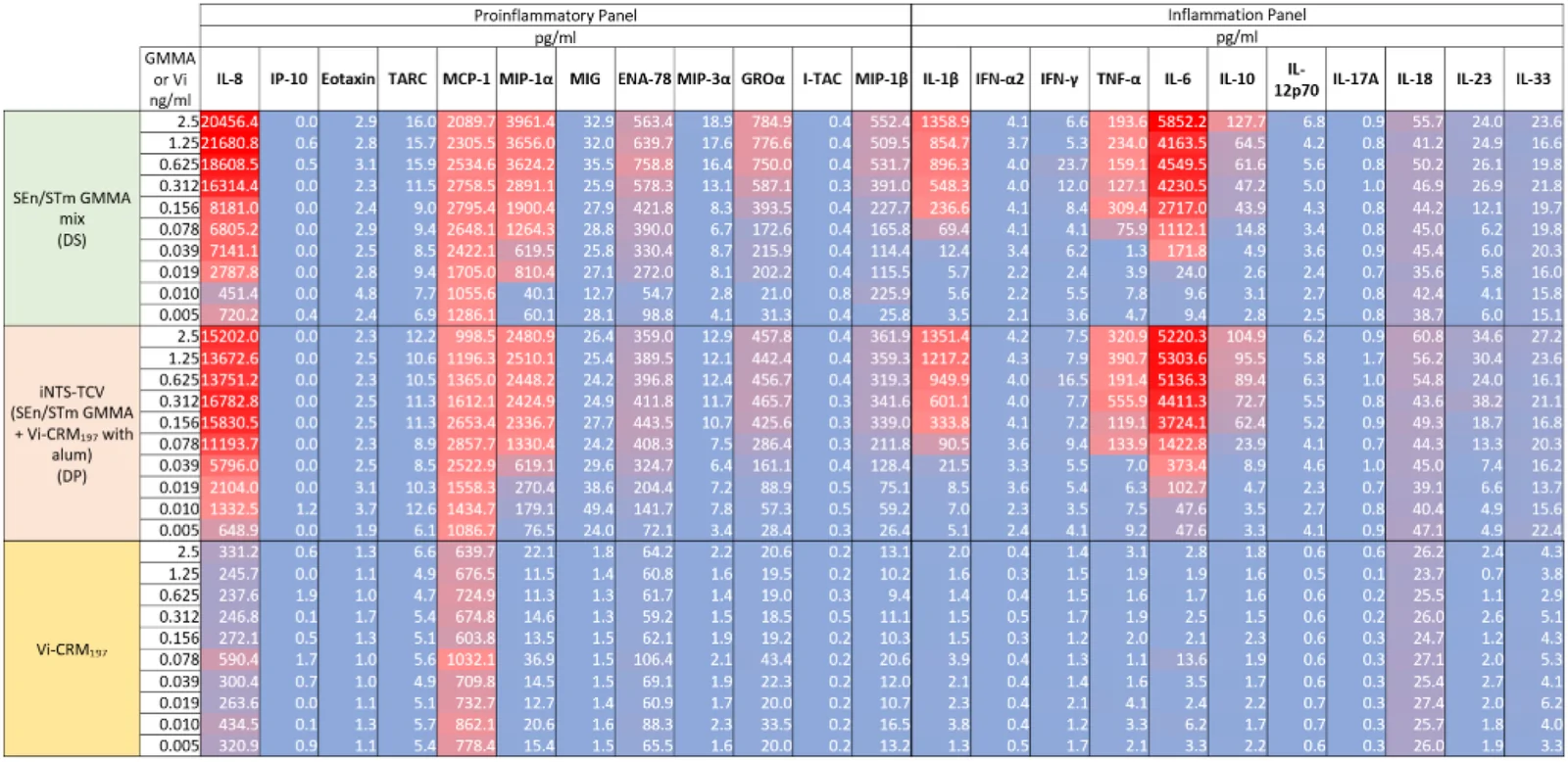
Heat map showing chemokine and cytokine expression by human PBMCs after incubation with a mixture of STmGMMA/SEnGMMA, or the iNTS-TCV vaccine, or Vi-CRM_197_ alone. Human PBMCs exposed to the iNTS-TCV vaccine elicited same chemokines and cytokines induction pattern than PBMCs exposed to an equal mixture of the STmGMMA and the SEnGMMA components. The Vi-CRM_197_ alone elicited less chemokines and cytokines induction. DS: drug substance; DP: drug product.

**Figure 4:**
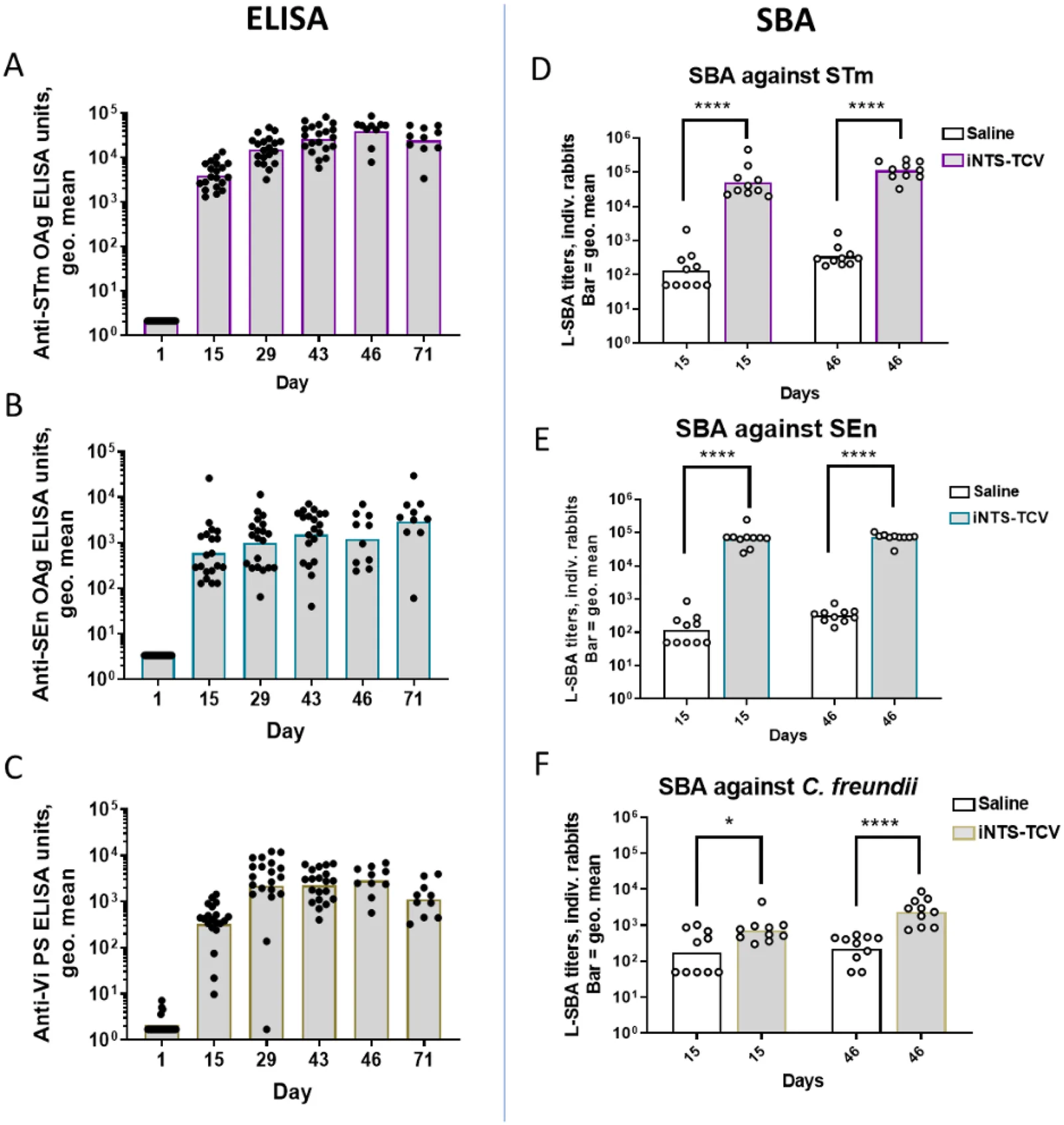
ELISA and SBA results from the repeated-dose toxicology study in New Zealand White rabbits. **(A–C) ELISA**: Anti-STm OAg (A), anti-SEn OAg (B) and anti-Vi (C) serum IgG measured in individual rabbits immunized with iNTS-TCV. Sera were obtained on Days 1, 15, 29, 43, 46 and 71. Black circles represent individual animals; bars indicate group geometric means. STm: *Salmonella enterica* serovar Typhimurium; SEn: *Salmonella enterica* serovar Enteritidis; OAg: O-antigen. **(D–F) SBA**: Serum bactericidal assay (SBA) IC50 values showing homologous bactericidal activity against *S*. Typhimurium D23580 (D), *S*. Enteritidis CMCC4314 (E), and *C. freundii* 3056 (F) at Days 15 and 46. White circles represent individual animals; bars indicate group geometric means. A nonparametric Mann-Whitney test was conducted to assess the statistical difference in the SBA response of sera from rabbits immunized with iNTS-TCV compared with sera from rabbis receiving saline (control). P-value annotation ‘ns’ refers to P > 0.05; * to P ≤ 0.05; and **** to P ≤ 0.0001.

**Table 1:** Comparative release testing of iNTS-TCV: non-GMP toxicology lot versus GMP lots from two different CMOs.

Test (Method)	Toxicology Lot LBP-5763 produced by GVGH. Batch size: 1L	Clinical Lot BK0121 produced by CMO-1. Batch size: 3L	Clinical Lot BK0123 produced by CMO-1. Batch size: 3L	Clinical Lot 20250402 produced by CMO-2. Batch size: 4L
Identification/ Quantification of SEn μg OAg/mL (FAcE)	Positive/40	Positive/41	Positive/35	Positive/40
Identification/ Quantification of STm μg OAg/mL (FAcE)	Positive/39	Positive/53	Positive/48	Positive/47
Identification/ Quantification of Vi polysaccharide μg/mL (HPAEC-PAD)	Positive/47	Positive/44	Positive/40	Positive/43
Free Vi (unconjugated) in the drug product % (HPAEC-PAD)	NA	8.8	8.0	5.0
GMMA not adsorbed to Alhydrogel measured by lipid A in drug product supernatant nmol/mL (HPLC-RP/MS)	0.2	0.3	1.2	0.4
Total Vi not adsorbed to Alhydrogel measured by 2-aminogalacturonic acid in drug product supernatant % (HPAEC-PAD)	61.0	71.6	69.0	67.0
Cytokine release by PBMC RPU/mL (MAT, Ph. Eur. 2.6.30, Method 2)	NA	1.2	1.6	1.3

SEn: *Salmonella enterica* serovar Enteritidis; STm: *Salmonella enterica* serovar Typhimurium; OAg: O-antigen; FAcE: Formulated Alhydrogel Competitive ELISA; HPAEC-PAD: High-Performance Anion-Exchange Chromatography with Pulsed Amperometric Detection; HPLC- RP/MS: High-Performance Liquid Chromatography – Reversed Phase / Mass Spectrometry; RPU: Relative Pyrogenic Units.

## Data Availability

Data supporting the findings of this study are available within the paper and its Supplementary Information.
